# The Analysis of Interpersonal Communication in Sport From Mixed Methods Strategy: The Integration of Qualitative-Quantitative Elements Using Systematic Observation

**DOI:** 10.3389/fpsyg.2021.637304

**Published:** 2021-03-31

**Authors:** Conrad Izquierdo, M. Teresa Anguera

**Affiliations:** ^1^Faculty of Psychology, Autonomous University of Barcelona, Barcelona, Spain; ^2^Faculty of Psychology, Institute of Neurosciences, University of Barcelona, Barcelona, Spain

**Keywords:** sport communication, interpersonal systems, mixed methods, quantitizing, systematic observation, analysis interaction, social contexts

## Abstract

The objective to which this manuscript is oriented to is focused on the analysis of interpersonal communication in sport. The multimodal essence of human nature adopts special characteristics in individual and team sports, given the roles that athletes adopt in different circumstances, depending on the contingencies that characterize each competition or each training session. The *mixed methods* framework allows us to advance in the ways of integration between qualitative and quantitative elements, taking advantage of the proven possibilities of systematic observation, which we can consider *mixed method* in itself, and which provides rigor and flexibility in the study of the communicative flow in sport. In any sport, the procedure followed by systematic observation may require direct observation, which is characterized by its high perceptiveness, or indirect observation, when it comes to verbal behavior or documentary material. In all cases, the procedure is structured in three macro-stages: QUAL-QUAN-QUAL. In this work we start from a conceptual positioning about interpersonal communication, to later show the sequential gear in sports about obtaining qualitative data, its transformation into other types of data that are still qualitative but have been structured, analyze them quantitatively, and return to a qualitative stage where the interpretation of the results is possible. This process of *quantitizing* constitutes the cornerstone that gives shape and structure to any research on interpersonal communication in sport that combines the fine nuances of qualitative data (a motor action, a gesture, an exclamation,…) with the power of robust quantitative data analysis suitable for the treatment of organized qualitative data, which will provide qualitative *feed-back*.

## Introduction

The conceptual and empirical scope of communication in sport is immense and undoubtedly polyhedral, and the unfolding of facets that are derived ranges from interpersonal communication to health, through humanistic, organizational and media approaches, until reaching the 22 sub-disciplines that Billings (2017) highlights, and that are grouped into four blocks: (1) how a certain subdiscipline of communication relates to sport, (2) specify the theories based on communication relevant to sport within the subdiscipline (3) search for jobs published that show the connection between sport and the subdiscipline of communication, and (4) articulate possible directions for future research within the framework of sport and the subdiscipline of communication.

To this we must superimpose the methodological plan, undoubtedly rich, which is fundamental in its analysis. In this context of diverse positions and dispositions that characterize the research agenda in communication and sport, the challenge of investigating interpersonal communication, incorporating traditional issues (vs. *media*) of social and organizational psychology is considered by Wenner (2015), as an outstanding provision to be developed under the name of *Communication Studies and Sport*. The objective of Wenner (2015) consists in highlighting the rich potential for an interdisciplinary fit between communication and sport, addressing new perspectives offered by interpersonal, group, and organizational interaction in sports contexts.

Communication and the relationships it generates are central in managing the perceptions of the various actors who have an active part in sport (athletes, coaches, managers, family members, doctors, physiotherapists, journalists, etc.) and taking into account the interrelation with one of the fundamental aspects, which is performance (Cunningham et al., 2018). According to Kassing and Matthews (2017), athletes are motivated by two-way communication, which is sometimes informal, and the results obtained show that their motivation is greater when the coaches give them support and take them into account. This would lead us to an interesting discursive perspective (Fairhurst and Putnam, 2004), referring to daily speech and the interactive network developed, including, of course, *online* communications. These, according to Riva (2002), consider an intergame from everyday interactions to reaching different communication levels, and depending on the organizational structures in which it is involved, considering, at the same time, that these constitute “a process constructed and enacted by its individual members” (Fielding-Lloyd and Meán, 2011, p. 346).

Sport is characterized by generating a complex multi-level network where competition, support, conflict resolution, and the creation of new contacts are continuously converged, which, in short, make up very diverse interpersonal relationships. In fact, sport itself, according to Turman (2017) can be considered as an interpersonal relationship, since it allows mediating relationships between athletes, coaches, families (in the case of children who practice it), etc.

The connection between the conceptual framework of interpersonal communication and research in the field of sport is extremely interesting, in order to know and evaluate the formation of these relationships, communicative styles, role played by messages, promotion of coach-athlete relationships, etc. In this sense, the influence that coaches generate on athletes is undoubted. Athletes must not only perform, but also understand and reflect on what the coach tells them (Turman, 2003). Despite the situation of “power”/power imbalance between athletes and coaches, the interaction is markedly interpersonal in nature, and to a large extent it tests coaches to gain complicity, persuade athletes, build a sincere relationship, and create a positive environment (Turman, 2017). This process is undoubtedly related to the prosociality between coaches and athletes (Turman and Schrodt, 2004). Likewise, the communicative style has a notable impact, and it is known from studies carried out (Turman and Schrodt, 2004) that the autocratic style of coaches negatively correlates with the affection that athletes show to sport, to their colleagues, and to the coach himself.

The interaction between coaches and athletes takes place in very different contexts (informal sessions, preparation for competitions, half-times, conversations in the locker room, etc.), which provides a wide range of communication possibilities and different types of messages (Turman, 2017) whose study is of great importance to delve into the analysis of interpersonal relationships between them.

From a conceptual perspective, according to Berger and Calabrese (1975) it is interesting to highlight the influence of the theoretical frameworks of social psychology and the convenience of working with theoretical proposals more focused on interpersonal communication processes. The necessary coexistence between the adopted theory and the implemented methodology leads us to point out that we have suitable theoretical frameworks. The construction of the relationship with the acquaintance (Newcomb, 1953), the aspects related to the perception of the person (Kaplan and Anderson, 1973) and the bases proposed by Berger and Calabrese (1975) on the reduction of the uncertainty in the development of interpersonal communication are proposals that fit with the systematic observation of the relationships that sport favors. Consider, for example, the communicative exchanges between athletes, around the behavior of the coach or with other social agents.

Likewise, this conceptual framework contemplates different levels of analysis, and in the same way that Knapp et al. (1973) studied the types of non-verbal behavior that occur in each phase of a communicative exchange, we can contemplate a strategy that allows a structured procedure, both *bottom up* (inductive path) and *top down* (deductive path). Both ways must be possible in the analysis of interpersonal communication structured as a problem of systematic observation.

The conceptual axes indicated and the foundations of the *development theory of interpersonal communication* (Berger and Calabrese, 1975), allow us to connect with the reality of interpersonal communication in sport, considering the possibilities that systematic observation offers, and smoothing out the difficulties posed by the traditional cracking between the radical qualitative vs. quantitative options, already denounced by Reichardt and Cook (1986), among many other authors, apart from other issues that are being developed in recent years, such as the compensation for the deficit of perceptivity in indirect observation (Anguera et al., 2018b; Anguera, in press, 2020).

Over four decades (1960-2000), most empirical studies carried out in all the sub-fields of the Social Sciences, and also, specifically, in interpersonal communication in sport, had been proposed following a qualitative or quantitative methodological orientation. This position was consolidated by each of the two options, which were in open confrontation, and radicalized over time. It was precisely from the beginning of the century and the millennium, around the year 2000, with variations according to the countries that mixed methods began to be put into practice. It initially implied a complementarity between the two perspectives (qualitative and quantitative), to finally evolve to an integration between qualitative and quantitative elements.

## Interpersonal Communication From Mixed Methods

In line with pragmatic thinking (Rorty, 1982), in the field of interpersonal communication there is no approach to the production and use of knowledge based on the conflict between methodological perspectives. Smith and Wilson (2010) have summarized the main epistemological and ontological assumptions that scholars of interpersonal communication take into account when working under the scientific umbrella of post-positivism also known as scientific realism (Pavitt, 1999), and that it is perfectly applicable to the field of sport. We refer to the dominant perspective (not the only one) of researchers in interpersonal communication as the empirical or post-positivist orientation based on a body of assumptions aimed at promoting: (a) the moderate position in the confrontation between empiricism and positivism; (b) the recognition of the role played by the opinions of the observer and the predictability of people's actions; (c) the distinction between causal logic and functional logic of theories; (d) the incorporation of the social and historical-cultural context; (e) the theoretical consideration of the sense of control that the social actor has over his life.

The refusal to take extreme positions on the side of empiricism or positivism and the acceptance that there is no single way of investigating because different questions require different answers are two features that help to reduce tension and avoid the rejection of the study of the more complex qualitative aspects of a socio-cultural nature that are part of the communicative interaction. What, then, is the position on the quantitative-qualitative dichotomy? Levine (2011) pointed out that the useful and valuable of both methodologies in the field of social sciences, and, therefore, in sport, is to raise a reasonable doubt about the veracity of the controversy between methodologies, holding that “… doing both qualitative and quantitative research well may be too ambitious for many mere mortals” (p. 28). Somehow we understand that in interpersonal communication in sport the approach of Mixed Methods (from now on MM) is not encouraged by the difficulty of training researchers in both paradigms (Anguera et al., 2017b, 2021a).

On the other hand, MM is a transdisciplinary methodological movement in which engaged researchers participate with social and behavioral studies (Creswell, 2016). The primary message or point of departure was to give visibility and awareness of a methodological practice, little taken into account when it comes to pointing out its background (Maxwell, 2016), based on the combined use of quantitative and qualitative data with independence of the positivist or interpretative position held by researchers (Creswell and Plano Clark, 2011).

Without denying or minimizing the urgent need to promote methodological training in mixed methods (Creswell and Plano Clark, 2011; Mertens et al., 2016) that is up to the new challenges of social research and behavior, the purpose of this work is precisely to show that systematic observation can structure the complexity of the empirical-analytical situation of communicative interaction in dyads and small groups (Anguera and Izquierdo, 2006; Sánchez-Algarra and Anguera, 2013), not only complying with the characteristics of the Mixed Methods methodology (Bazeley, 2018), that require evidence on the integration of qualitative and quantitative elements, but also making its own and specific contribution, precisely because, in itself, it constitutes a mixed method (Anguera and Hernández-Mendo, 2016, 2019; Anguera et al., 2017a), throughout its QUAL-QUAN-QUAL macro stages.

If our way of synthesizing the position of scientists, affiliated with the fields of interpersonal communication or mixed methods, is considered correct, we can fully incorporate these two independent but related considerations about the coexistence of different methodological perspectives: first, (i) the good practice of researchers committed to qualitative or quantitative methodology does not require them to choose between a certain line of methods and techniques (Creswell and Plano Clark, 2011; Levine, 2011) and, secondly, (ii) the criterion of flexibility and methodological adaptation takes precedence over time to make decisions in favor of a methodological approach that qualitatively and quantitatively structures the complexity of the object of study (Anguera et al., 2018a).

According to these two statements, first of all, we address a brief general overview on the programmatic proposal of the MM methodology promoted by the MMIRA (Mixed Methods International Research Association), whose most relevant distinctive note is the integration of elements: data, results, procedures,…, qualitative and quantitative (Mertens et al., 2016). The next step has to do with the options of observing, measuring and evaluating human communication processes. In line with our interests, the selected methodological perspective is the analysis of the interaction (Poole and McPhee, 1985; Tardy, 1988; Brauner et al., 2018) focused on the studies that formalize and compute the nominal data that inform about the dynamics of the current interpersonal communication observed in sport. Finally, the observation process linked to the analysis of the interaction is deepened, adapted and extended to new approaches on how to obtain a large quantity of qualitative data and be able to manage, quantify and analyze ensuring the rigor of the entire process. Specifically, the methodological guide for systematic observation is synthesized (Anguera and Blanco-Villaseñor, 2001; Portell et al., 2015a,b; Anguera et al., 2018b) pointing out the strengths of the observational methodology and the dispositions that are taken to deal solvency with the weaknesses associated to the non-existence of measurement instruments or standard qualifiers, and the potential biases attributable to the behavior of the observer-coder. We conclude by summarizing the permeability and robustness attributes of the systematic observation considered as a suitable and good strategy of full integration in MMR.

## Integration in Mixed Methods Research: Multi-Paradigmatic Matrix

The concern for epistemic and social quality also defines the fundamental axes that move the discourse of the MM methodological perspective (Mertens et al., 2016): (i) the requirement of rigor in the design of interpersonal communication studies in sport applied to a wide variety of problems detected or expressed in the sport communities, and (ii) the scientific requirement to be able to share the validity of the new knowledge obtained, which involves reproducing results of the research and transferring them to different contexts in the field of sports.

To this day, the MM community (Creswell, 2016; Mertens et al., 2016) recognizes having identified the main research designs, the variants used and their application procedures. Hence, we can verify the presence of solvent works in some areas, and specifically in sport. However, the increase in examples of validated designs with postmodern and emancipatory sensitivity is lacking (Creswell and Plano Clark, 2011). It follows, therefore, that the bulk of the MM scientific production moves in the more pragmatic plane of the combination-integration of quantitative and qualitative data (Johnson and Onwuegbuzie, 2004; Teddlie and Tashakkori, 2012; Bazeley, 2018). In addition, the problem of the evaluation of MM quality is a core issue (Fàbregues and Molina-Azorín, 2017).

The sustained effort to define the MM methodology in accordance with the issues that the most influential voices are raising at different moments of its trajectory has led (Johnson et al., 2007) to point out some currently shared reference points by the members of the MMIRA.

MM research, committed to social reality, seeks to enhance the quality standards of scientific research through the proposal of new evaluation designs (Greene et al., 1989; Greene, 2006, 2007) that combine different methodologies. The multi-paradigmatic dialogue feeds mainly (Shannon-Baker, 2016) of the pragmatic mentality of dialectical pluralism, which respects the variety of points of view about the world and the ways of knowing reality (Onwuegbuzie and Frels, 2013), and critical realism (Maxwell and Mittapalli, 2010), which puts the accent on the partiality of knowledge, the importance of context, and on the influence of emotions, beliefs and values given that they are also part of this reality.

**Figure 1:**
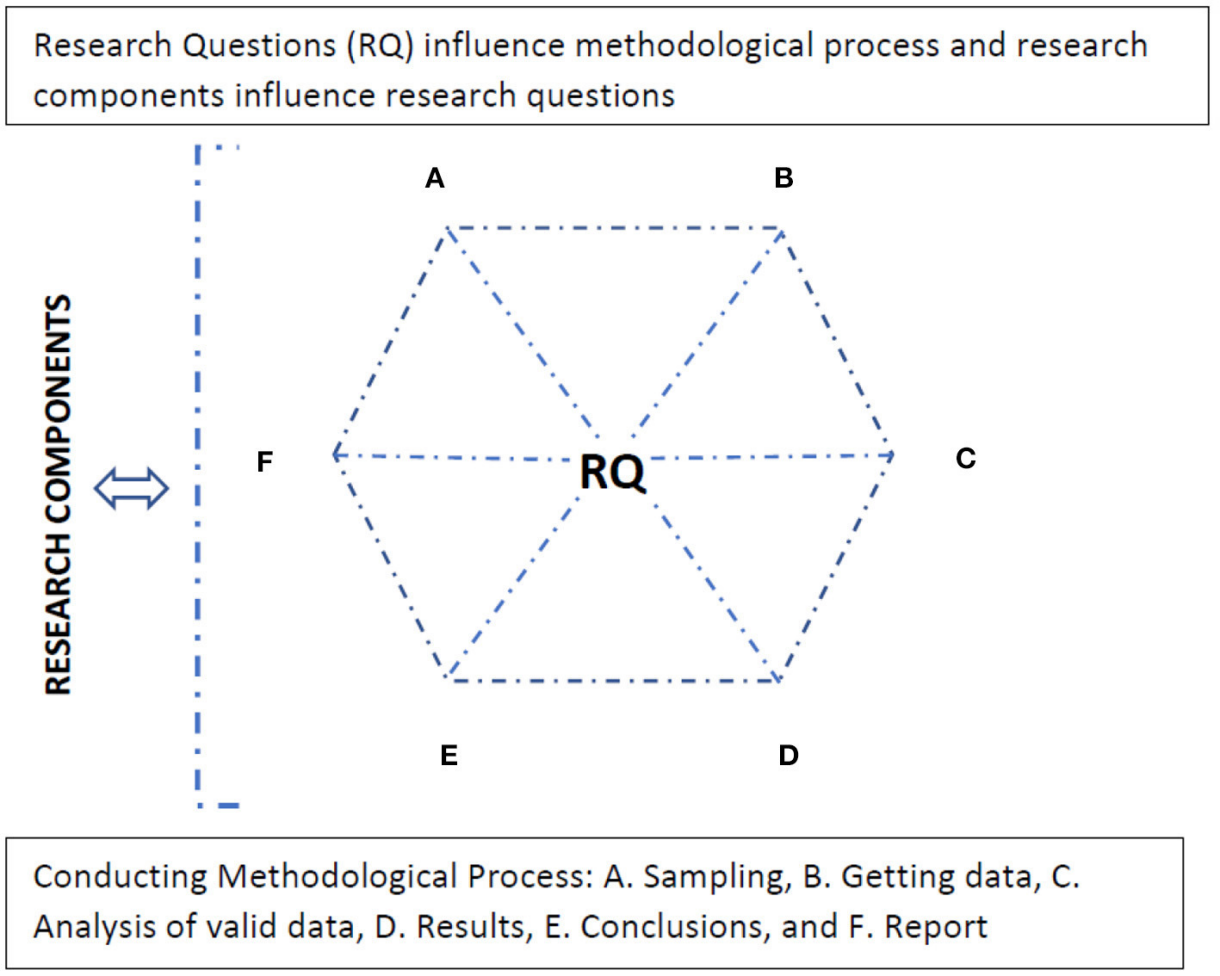
Reciprocal relationship between Research Questions and Methodological Process.

The MM integration emphasizes the central role of the research questions (Figure 1) when considering that they are present in all the related phases of the methodological process and that the definition of the research problem through the questions that are posed is, in turn, directly related to the central and guiding components of a rigorous scientific study: purposes, theories and beliefs, methods and validity considerations (Mertens et al., 2016). In other words, the object of study, which is interpersonal communication in sports, determines the methodology, and the methodology structures the object of study.

## Systematic Observation and Interpersonal Communication in Sport

**Figure 2:**
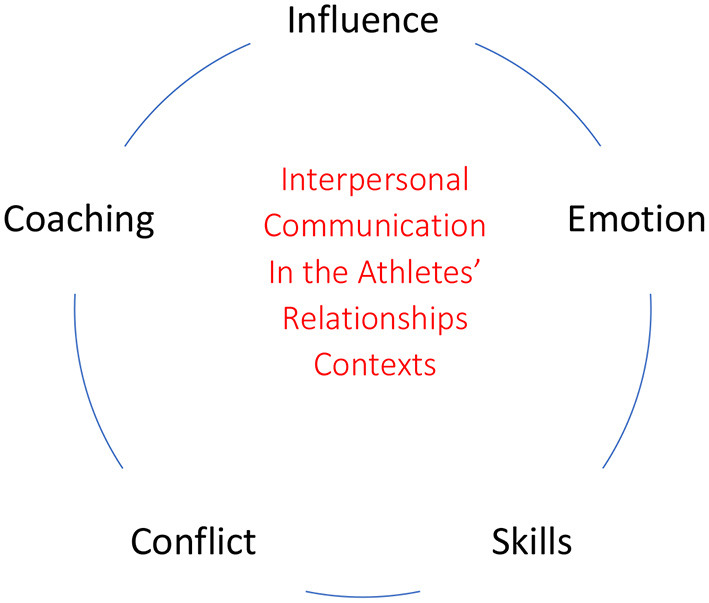
Main keywords for interaction analysis through systematic observation.

Communication in sports, an area that deserves full attention as an essential component of the fabric of inter and intra organizational relationships (Pedersen et al., 2020), includes two basic questions: what interlocutors do and say and how they interdependently weave domithe communications that keep them engaged in conversational activity until their closure. With the term “interaction analysis” we are referring to a grouping (cluster) of methods, techniques and perspectives that have in common the identification, in their interactive context, of sequences, patterns, uses of language, and so on, from the obtaining of preferably observational data. Regarding the constellation of key terms that can guide the realization of research problems, Figure 2 lists five interpersonal communication processes that can be structured as systematic observation problems in the field of communication and sport studies.

The conversational encounter between athletes and coaches, between athletes, between athletes and their families, between athletes and other social agents, etc., involves a visible and/or audible interaction, direct, either face-to-face or mediated, or indirect if the exchange is deferred (Poyatos, 1983). To refer to the interpersonal communicative encounter properly we adopt the expression “face-to-face interaction,” highlighting the idea that the behavior manifested in both poles of the interaction can be understood as a dimension (and potential subdimensions) that admits different degrees of activity according to the interpersonal situation that is studied from the individual reading of a book to daily formal and informal conversations through exchanges of written messages or voice notes (Nunes, 2020; Nunes et al. in review).

We will consider the methods of analysis of the interpersonal communication supporting an objective-negotiated vision of reality (not all representations of reality are equally acceptable) that structures the quantitative methodology committed to the social meaning and the meticulous control of the inference (Kendon, 1990; Bavelas et al., 2002; Levine, 2011; Krippendorff, 2013).

Gathering the elements that we have just enumerated, we understand as interpersonal communication analysis (Bakeman and Gottman, 1997; Keyton, 2018) the systematic observation study focused on the dynamic process of exchanges of interdependent behaviors (or actions) between two or more people with the purpose of classifying them, identifying their functions in the discourse or its structures (sequences, patterns, repetitive cycles,…), including the context in which the verbal and non-verbal interaction is developed: interpersonal conflicts, classroom, marriages, family, psychotherapy, work meetings, etc.

Obtaining observational data involves segmenting the stream of interpersonal communication behavior into units, classifying them according to a set of discrete categories and recording the order or sequence in which the behaviors occur. The instrument of observation is conceived as a system for channeling information, which the researcher must adapt, if he borrows it, or build it *ad hoc* according to the research problem addressed.

The necessary rigor in the process of obtaining observational data requires being able to verify its reliability in two different planes: on the one hand, the coders have to agree and be consistent in the procedure to be followed (unitizing reliability) and, on the other hand, the encoders will also have to show agreement in the classification given to the units (classificatory reliability). Reliability understood as agreement between observers is commonly tested with Cohen's kappa statistic (Cohen, 1960) or other widely known and used coefficients -in addition to those collected in the HOISAN free program (Hernández-Mendo et al., 2012). Data quality can be compromised by omission and commission biases introduced in the individual use of the categories. It is advised (Bakeman and Gottman, 1997) to calculate agreement coefficients for each individual category in addition to obtaining the overall coefficient of the instrument used.

In terms of validity (Rogers and Millar, 1982; Tardy, 1988), we must take into account what the researcher wants to know about the interaction under study: (i) verify a theory with the observational data obtained without including the interpretations of the participants (observer-privileged or experienced mode); (ii) use a coding system to represent the shared meanings of interpersonal communication between members of a culture (generalized subject-privileged or experiencing mode); (iii) identify the idiosyncratic meaning of the interaction that people who share a particular relationship have (restricted subject-privileged or experiencer mode). These three modes of observation require different types of evidence.

## Qual-Quan-Qual Integration Applying Systematic Observation Methods

The processes of systematic observation that make up the analysis of interpersonal communication have been deepened and enriched in the last 50 years, providing a neo-positivist vision sensitive to the social burden of behavioral events while proposing methodological controls, incorporating new technologies, developing powerful analysis techniques, and theorizes and protocols the procedure of observation as a methodological option in the strict sense, and this fit is optimal in the field of sport.

Creswell and Plano Clark (2011, p. 7) affirmed: “There are three ways in which mixing occurs: **merging** or converging the two datasets by actually bringing them together, **connecting** the two datasets by having one build on the other, or **embedding** one dataset within the other so that one type of data provides a supportive role for the other dataset” (highlight is done by us). This appointment has a fundamental relevance, which is still increasing because it has been backed by practically all the relevant MM researchers.

Indeed, of the three forms of integration that schematize, merging is usually used when initially or at successive moments of time we have qualitative and quantitative information; connecting when the data is processed, among the many possibilities available; and embedding when a minority data type is nested in data of a different nature, and predominantly.

The process of systematic observation is perfectly located in the connecting, given that the scientific procedure that supports it corresponds to the three major stages QUAL-QUAN-QUAL, and between the first two is where *quantitizing* is located, so much studied today (Anguera et al., 2020).

**Figure 3:**
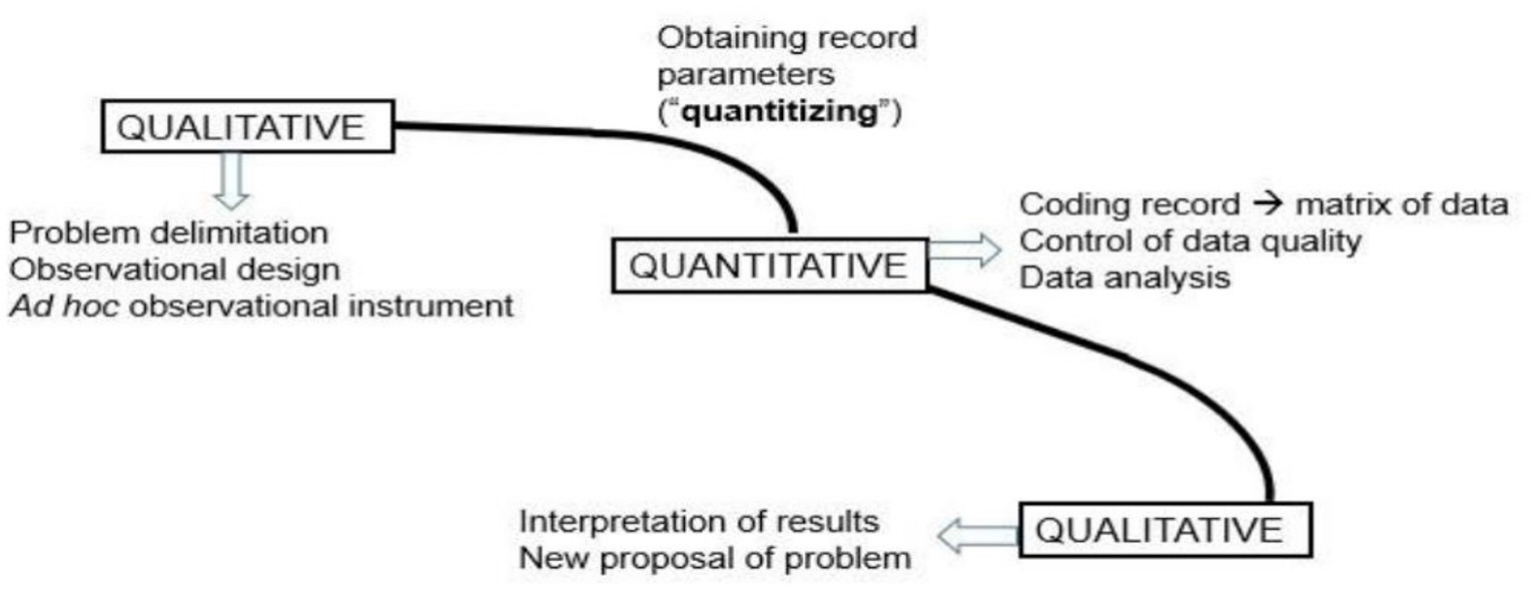
Macro steps of systematic observation process: QUAL-QUAN-QUAL.

In the first place, and in the field of interpersonal communication, we graphically show the three major stages in the procedure of systematic observation, which is a scientific procedure, but with some peculiarities that characterize it (Figure 3).

**Figure 4:**
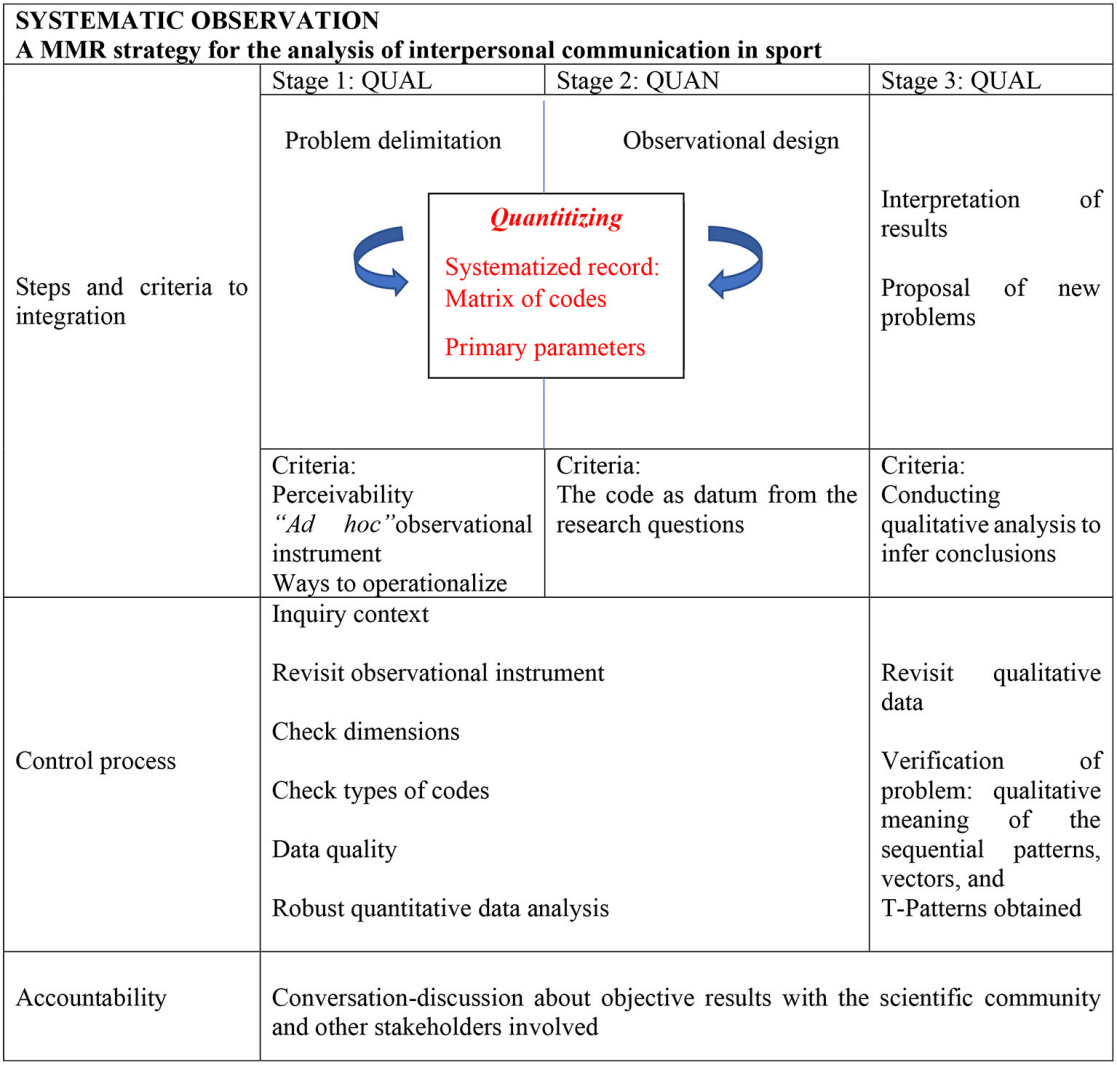
Integrating data, *Quantitizing*, and analysis in systematic observation: Steps (row 1), Control process (row 2), and Accountability (row 3).

We are particularly interested in highlighting the quantitizing focus, between the QUAL and QUAN stages, about which much has been written in recent years. Prestigious mixed methods authors have referred conceptually, and also technically, to quantification (Creswell et al., 2003) and to everything that involves the transformation of data (Sandelowski et al., 2009). But the great difference with observational methodology (Portell et al., 2015a,b), in favor of this, is that the quantification is more robust, since it is not only based on the counting of behavior occurrences, that is, in the frequency, but in the other primary parameters (Bakeman, 1978; Sackett, 1987; Bakeman and Gottman, 1997; Anguera and Blanco-Villaseñor, 2001; Bakeman and Quera, 2011) of order and duration, which present a progressive order of inclusion. This quantitizing is situated between the first qualitative and quantitative macro-stages, and is produced thanks to the matrix of codes constructed from the sequence of co-occurrences (Figures 3, 4).

The transformation of the qualitative record register is possible both in direct observation (Sánchez-Algarra and Anguera, 2013) and indirect observation (Anguera et al., 2018b), and both are possible in interpersonal communication in sport. In many fields of interpersonal communication application, such as in clinical psychology (Roustan, 2010; Roustan et al., 2013; Anguera, 2017; Arias-Pujol and Anguera, 2017, 2020; Del Giacco et al., 2019, 2020; Anguera, in press, 2020), or in educational psychology (Tronchoni et al., 2018; Belza et al., 2019a,b; Escolano-Pérez et al., 2019; Sagastui et al., 2020), or in family psychology (Cuervo, 2014), the focus on verbal, or vocal, or both behavior is becoming more interesting, so that one more step of the procedure is the transformation of the registry in a code matrix.

In direct observation (Anguera, 2017), the modus operandi is already established, and for some years quantitative analysis of qualitative data has been carried out (mainly lag sequential analysis, polar coordinate analysis of polar, detection of T-Patterns) (Anguera et al., 2021b) which have been systematized according to the code matrix format, where the columns correspond to the dimensions or sub-dimensions of the observation instrument developed *ad hoc*, and the rows correspond to each of the observation units. The connecting is operating, through the transformation of the qualitative data that make up the record. It is an indisputable integration, given that we operate quantitatively on data that is textual, which we have only organized, and are essential when we start with individual cases and we are interested in accessing a hypothetical multiple case. It does not follow the proposals of Sandelowski et al. (2009), but we attest to its effectiveness, which we have repeatedly tested.

In indirect observation, obviously, greater methodological controls are required, in order to avoid the risk of inference, but the path to follow is also configured (Anguera et al., 2018b; Anguera, in press, 2020). The most complex issues are in the construction of the indirect observation *ad hoc* instrument, and in the application of the text segmentation criterion in units. In indirect observation we intend the “liquefying” of the text (resulting from the transcription of the communicative flow), which although it can be considered arduous depending on the theoretical frame of reference, and the contextual environment in which it is carried out, offers great methodological possibilities, with a certainly innovative cutting, as is the use of codes that have been obtained in the registry -even in programs of indirect observation (Anguera, in press, 2020). For example, if the ATLAS.ti program has been used, all the codes that appear in the window on the right are arranged in the form of a code matrix, so that the beginning of each row corresponds to the beginning of the text unit. In the studies carried out, we know that the quantitizing has been able to be carried out without problem from the text, and then applying a T-Patterns technique (Magnusson et al., 2015; Anguera et al., 2018a; Casarrubea et al., 2018) or applying sequential analysis of delays and analysis of polar coordinates (García-Fariña et al., 2018).

To continue to guarantee *scientific accountability*, the QUAL-QUAN-QUAL conversion must engage and shape the process of inferring, discussing and concluding the interpretation of results. The interpretation must be qualitative and based on the communicative acts and their consequences (*how the participants do social actions and what for… in its immediate communicative context*, Bavelas et al., 2002, p. 110) on the results obtained through robust quantitative analysis of the data sequences recorded in the studies of direct or indirect observation of interpersonal communication in sport.

In effect, the interpretation of communicative behavior (Poyatos, 1983; Bavelas and Chovil, 2000) is sustained by assuming the intentional and inferential character of social interaction in the communicative context of exchanges (Bateson, 1972). Other fundamental aspects of interpersonal communication are variability, cohesion, and coherence with the context (interview, personal papers, reports, rating scales, etc.).

On the other hand, the micro-analytical record of visible and audible communicative interpersonal behavior (stages 1 and 2 of the *quantitizing* process) that includes the use of highly elaborate notational systems (Anguera and Izquierdo, 2006; Izquierdo and Anguera, 2018), makes it possible to identify the linguistic resources of the interlocutors when they have a common or specialized exchange. For example, the linguistic movement markers that correspond to fleeting comments produced from the listener position, the turn and turn change markers, expressive communicative acts, symbolic gestures performed with the body, socio-affective adjectives, etc.

Regarding the interpretation of the results that integrate the quantitative and qualitative analysis within the scientific community in the field of sport, the similarities and disparities that other authors have obtained should be commented on, making a substantive and methodological self-criticism of the work carried out, and suggesting those elements that may lead to a continuation of the investigation.

We understand the power of the process of the three QUAL-QUAN-QUAL macro-stages, summarized in Figure 4, that fortify the *mixed methods* approach. Namely, the initially qualitative information obtained in the record, which is extremely rich for the intended purpose, allows a wide range of quantitative and qualitative analysis-interpretation, integrating both.

## Conclusion

In this work we have set out to study interpersonal communication in sport, taking advantage of the wide opportunities offered by systematic observation, as a scientific procedure that guarantees objectivity and rigor in the various stages of this procedure. For its materialization, we have focused on the systemic purpose of integrating qualitative and quantitative data forming transparent, rigorous and committed research designs with knowledge (exploratory and explanatory) and decision making (based on formal evaluations). The complexity of the interpersonal communication situations of daily life in different sports agents (athletes, coaches, families, managers, physiotherapists, journalists, etc.) and the important weight arried by the necessary incorporation of different data sources and analysis techniques, has led researchers in mixed methods to propose successive approaches to link the different methods included in the MM designs, from the distinction between component designs and integrated designs, through the four families of concurrent, sequential, conversion, and fully integrated designs, up to the recent reflection on the integration of the data and the results obtained with the MM methodology (Maxwell, 2013, among other contributions). We have incorporated as a reference for the MM framework the reflection that argues the complete integration of qualitative and quantitative elements. We have approached the exhibition taking as a starting point the consideration that systematic observation is a positive scientific methodology in the strict sense, with an application protocol that covers all the components of the scientific method, regulates the conduct of the methodological process and promotes commitment to quality controls and good research practice.

The attribute of permeability is part of the process that we have just defined and that crystallizes in a deep elaboration of the units of observation, recording and interpretation. Without this formalizing step of the units, the quantitization would lose its operative force as a connecting element between the macro-stages of the systematic observation process. From a systemic point of view, the need for this synergistic connection emerges as a result of the reallocation of permeable boundaries between the qualitative and quantitative elements (Sánchez-Algarra and Anguera, 2013; Anguera et al., 2017b, 2018b).

We are convinced that our work will allow interpersonal communication scholars in sport and MM researchers to consider the possibilities and rigor offered by the observational methodology conceived as an integrated QUAL-QUAN-QUAL process applied to the analysis of communicative interaction in a wide range of sports situations.

## Author Contributions

All authors listed have made a substantial, direct and intellectual contribution to the work, and approved it for publication.

## Conflict of Interest

The authors declare that the research was conducted in the absence of any commercial or financial relationships that could be construed as a potential conflict of interest.
